# Protective and Pathogenic Roles of CD8+ T Lymphocytes in Murine *Orientia tsutsugamushi* Infection

**DOI:** 10.1371/journal.pntd.0004991

**Published:** 2016-09-08

**Authors:** Matthias Hauptmann, Julia Kolbaum, Stefanie Lilla, David Wozniak, Mohammad Gharaibeh, Bernhard Fleischer, Christian A. Keller

**Affiliations:** 1 Department of Immunology, Bernhard Nocht Institute for Tropical Medicine, Hamburg, Germany; 2 Institute for Immunology, University Medical Center Hamburg-Eppendorf, Hamburg, Germany; Mahidol University, THAILAND

## Abstract

T cells are known to contribute to immune protection against scrub typhus, a potentially fatal infection caused by the obligate intracellular bacterium *Orientia (O*.*) tsutsugamushi*. However, the contribution of CD8+ T cells to protection and pathogenesis during *O*. *tsutsugamushi* infection is still unknown. Using our recently developed BALB/c mouse model that is based on footpad inoculation of the human-pathogenic Karp strain, we show that activated CD8+ T cells infiltrate spleen and lung during the third week of infection. Depletion of CD8+ T cells with monoclonal antibodies resulted in uncontrolled pathogen growth and mortality. Adoptive transfer of CD8+ T cells from infected animals protected naïve BALB/c mice from lethal outcome of intraperitoneal challenge. In C57Bl/6 mice, the pulmonary lymphocyte compartment showed an increased percentage of CD8+ T cells for at least 135 days post *O*. *tsutsugamushi* infection. Depletion of CD8+ T cells at 84 days post infection caused reactivation of bacterial growth. In CD8+ T cell-deficient beta 2-microglobulin knockout mice, bacterial replication was uncontrolled, and all mice succumbed to the infection, despite higher serum IFN-γ levels and stronger macrophage responses in liver and lung. Moreover, we show that CD8+ T cells but not NKT cells were required for hepatocyte injury: elevated concentrations of serum alanine aminotransferase and infection-induced subcapsular necrotic liver lesions surrounded by macrophages were found in C57Bl/6 and CD1d-deficient mice, but not in beta 2-microglobulin knockout mice. In the lungs, peribronchial macrophage infiltrations also depended on CD8+ T cells. In summary, our results demonstrate that CD8+ T cells restrict growth of *O*. *tsutsugamushi* during acute and persistent infection, and are required to protect from lethal infections in BALB/c and C57BL/6 mice. However, they also elicit specific pathologic tissue lesions in liver and lung.

## Author Summary

*Orientia (O*.*) tsutsugamushi* is the causative agent of scrub typhus, a potentially fatal disease that is endemic in South East Asia. This bacterium replicates in the cytoplasm of its host cells. The obligate intracytoplasmic lifestyle resembles that of many viruses, but among pathogenic bacteria it is unique to *O*. *tsutsugamushi* and the closely related *Rickettsia* spp. CD8+ T cells are specialized on the recognition of cytoplasm-derived antigens and are therefore important in antiviral and antitumor immunity. Using two different mouse models, we show that CD8+ T cells protected against lethal outcome of *O*. *tsutsugamushi* infection. Moreover, CD8+ T cells were implicated in the development of tissue lesions in liver and lung. Mice that lack CD8+ T cells due to a genetic defect developed a massively increased macrophage response that failed to control the infection. In protected wildtype mice, the CD8+ T cell-driven immune response elicited the recruitment of macrophages to distinct locations in liver and lung. We also show that CD8+ T cells were important to prevent replication of *O*. *tsutsugamushi* many weeks after the recovery from any signs of disease. Therefore we propose that a well-balanced relation between pathogen burden and a potentially harmful CD8+ T cell-dependent immune response becomes established during infection with *O*. *tsutsugamushi*.

## Introduction

*Orientia (O*.*) tsutsugamushi*, the causative agent of scrub typhus, is an obligate intracellular bacterium of the Rickettsiaceae family. The pathogen resides in the cytoplasm of its host cells, which are mainly macrophages, monocytes and dendritic cells (DCs) [1, 2]. Furthermore, infection of cardiomyocytes, hepatocytes and epithelial cells has been reported [3–5]. Infection of endothelial cells is a hallmark of most *Rickettsia* species and can be seen in mice infected with *O*. *tsutsugamushi* by the intravenous route [6] and in severe human cases of scrub typhus [3, 7].

Protective immunity against *O*. *tsutsugamushi* is believed to depend on cellular immunity with interferon (IFN)-γ being the key mediator [8–11]. Data from *in vitro* studies suggest that activated macrophages contribute to intracellular killing of *O*. *tsutsugamushi* [12, 13].

CD8+ T cells are important effectors against pathogens that reside in the cytoplasm of their host cells. Rollwagen et al. gave a first hint that cytotoxicity might play a role in anti-*Orientia* immunity by demonstrating that splenocytes from infected mice lyse *O*. *tsutsugamushi*-infected MHC-matched L929 mouse fibroblasts *in vitro* [14]. De Fost et al. later showed that granzymes are upregulated in lymphocytes of scrub typhus patients, suggesting that cytotoxic cells play a role in anti-*O*. *tsutsugamushi* immunity in humans [15].

Studies on T cells in human scrub typhus patients describe very well the composition and phenotypic characterization of T cells in the peripheral blood [16]. Functional studies on the contribution of T cell subpopulations and T cell dependent effector mechanisms in *O*. *tsutsugamushi* infection however are missing so far.

While many bacterial pathogens are eradicated from the body by the host’s immune response after an acute infection, there is increasing evidence that *O*. *tsutsugamushi*, similar to other members of the Rickettsiaceae family like *R*. *prowazekii*, can persist for many months or even years in quantities that are below the detection limit of state of the art techniques [17]. Remarkably, the hosts in persistent Rickettsia infections appear healthy. Persistent *O*. *tsutsugamushi* infections were reported in humans as well as in mice [18–21]. Reactivation of the infection could be observed after infection with a heterologous strain or upon treatment with the immunosuppressant cyclophosphamide (CP) [20]. So far, the cellular mechanism that controls low-level *O*. *tsutsugamushi* infection has not been further elucidated: CP is an immunosuppressant with a broad target cell range that includes mature hematopoietic progenitors and all lymphocyte subsets; it acts as an alkylating compound that crosslinks DNA and is cytotoxic to cells expressing low levels of aldehyde dehydrogenase [22–25]. It was therefore of interest to study whether CD8+ T cells play a role in controlling low-level infection during latency.

Mice belong to the natural host range of *O*. *tsutsugamushi* [26, 27] and have long been used as model animals, using subcutaneous (s.c.), intraperitoneal (i.p.), intravenous (i.v.) and intradermal (i.d.) inoculation routes [6, 28–31]. However, the wide range of clinical outcomes of *O*. *tsutsugamushi* infection in humans cannot be recapitulated in a single mouse model. Each model has specific advantages and limitations. The footpad infection model [1] that involves inoculation via the dermis [32, 33] closely mimics the natural inoculation route during chigger feeding. Moreover, it recapitulates many other features of human infection, including regional lymphadenopathy [34], bacteremia and systemic cytokine response [35], macrophage/monocyte tropism [2], and a multi-organ involvement with similar patterns of pulmonary [36, 37] and hepatic inflammation [4, 38, 39]. As for the differences to human infection, it has to be noted that footpad inoculation does not result in eschar formation [1]. Endothelial infection, which is found primarily in lethal human cases [2, 3] is absent in footpad-infected mice. Also, footpad infection with *O*. *tsutsugamushi* is uniformly self-healing in C57BL/6 and BALB/c mice, thus modeling the 70–100% of untreated human cases that survive [40]. For investigation of other aspects of *O*. *tsutsugamushi* infection, e.g. endothelial dysfunction, renal injury [41] or immunopathology mediated by host factors [42], other inoculation models may be more suitable.

If CD8+ T cells played a non-redundant role in *O*. *tsutsugamushi* infection, their absence–as in antibody-depleted or genetically CD8+ T cell-deficient mice–would result in loss of the self-healing, protective phenotype. Concomitantly, we used i.p. infection with the Karp strain of *O*. *tsutsugamushi* [31] in order to study immune-mediated protection to an otherwise rapid lethal infection.

In this study, we demonstrate the importance of CD8+ T cells in the acute and persistent phases of an *O*. *tsutsugamushi* infection, and also show that CD8+ T cells contribute to tissue injury.

## Materials and Methods

### Mice

All *in vivo* experiments were carried out at the BSL3 animal facility of the Bernhard Nocht Institute for Tropical Medicine in Hamburg. Female 5–9 week-old C57BL/6N or BALB/c mice were purchased from Charles River (Sulzfeld, Germany). Male C57BL/6-beta 2-microglobulin^-/-^ (β_2_m^-/-^) mice and their respective C57BL/6 wildtype control animals were kindly provided by Caroline Johner (MPI, Freiburg, Germany). Male CD1d^-/-^ mice, Prf1^-/-^ mice, and their respective C57BL/6 wildtype controls were bred at the animal facility of the Bernhard Nocht Institute for Tropical Medicine. Food and water was provided ad libitum. Six to ten weeks old mice were infected, unless otherwise stated, with 5×10^3^ spot-forming units (sfu) of an *O*. *tsutsugamushi* Karp inoculum consisting of infected, irradiated L929 cells, by a combined subcutaneous (s.c.) and intradermal (i.d.) injection into the right hind footpad, or by intraperitoneal (i.p.) injection [1]. Mock-infected control groups received irradiated L929 cells via the same injection route. The *O*. *tsutsugamushi* Karp strain is an endemic human pathogenic strain [43] that has been largely characterized in previous studies by others and by us [1, 6, 31, 44].

Mice were sacrificed by CO_2_-narcotization followed by cervical dislocation. Lung perfusion (described below) however was performed under irreversible anesthesia given through i.p. injection of high-dose ketamine and xylazine. Mice euthanized for reasons of animal welfare, were considered as deceased for statistical analyses.

### PCR

Bacterial organ loads and bacteremia were measured by a multicopy *traD* qPCR, as described previously [1]. 10 ng or 20 ng DNA were used in each PCR-reaction; for subsequent statistical analyses, results were transformed as *y = log*_10_(100*×copies+*1) or *y = log*_10_(50*×copies+*1), respectively. Graph scales indicate the 10^y^-values.

### Preparation of cell suspensions and flow cytometry

20 μl of peripheral blood were collected from the tail vein and collected in FACS buffer. Splenocytes were prepared by grinding spleens between frosted-end microscopic slides in petri dishes containing RPMI medium supplemented with 5% FCS. For analysis of pulmonary cells, mouse lungs were perfused through the pulmonary artery with 5 ml PBS. The lungs were excised, cut into small pieces and incubated for 60 min at 37°C in RPMI medium supplemented with 5% FCS and 200 μg/ml Collagenase D (Roche Diagnostics, Risch, Switzerland) and 10 μg/ml DNAse I (Sigma, Deisenhofen, Germany). The suspension was resuspended with a Pasteur pipette every 15 min. The reaction was stopped by 5 min incubation at 37°C with 5 mM EDTA. Lung cells were then mechanically separated in 100 μm mesh sized cell strainers.

Erythrocytes were lysed with Tris-buffered 0.15 M ammonium chloride for 1 min (lung) or 7 min (blood, spleen). Unspecific binding sites were blocked with anti-FcγR at 4°C for 10 min. The cells were extracellularly stained with anti-CD4-FITC, anti-CD4-APC, anti-B220-APC, anti-CD3-PE, anti-CD8-PerCP-Cy5.5, or anti-CD62L-FITC (all from BD, Heidelberg, Germany), respectively, for 1 h at 4°C. Cells were analyzed on an Accuri C6 cytometer or fixed in 1% PFA for 30 min and analyzed on an LSR-II cytometer (both BD). Absolute cell numbers of blood cell populations were measured with the C6 cytometer.

### Depletion of CD8+ T cells

YTS169 Hybridoma cells (kind gift from H. W. Mittrücker, University Hospital Eppendorf, Hamburg, Germany) producing monoclonal antibodies against CD8 were cultivated in protein-free hybridoma medium (PFHM-II; Gibco, UK). Antibodies were precipitated in ammonium sulfate and dialysed in PBS. For depletion of CD8+ T cells, 0.5 mg/ml of YTS169 was injected i.p. at indicated time points. For CP treatment, 8 mg of CP were injected i.p. followed by a second dose of 4 mg CP 4 or 5 days later. Depletion efficiency was measured by flow cytometry of antibody-stained peripheral blood leukocytes (see S1 Fig for depletion during acute phase; S2 Fig for depletion during persistent phase).

### Adoptive transfer

Splenocytes were isolated from *O*. *tsutsugamushi*-infected mice or from control mice. CD8+ T cells were then isolated by negative magnetic selection, using the CD8+ T cell isolation kit (StemCell Technologies, Vancouver, Canada). Magnetic separation resulted in more than 75% purity of CD8+ T cells with less than 5% contaminating CD4+ T cells (S3 Fig). 1×10^7^ cells were then injected intravenously (i.v.) into naïve recipient mice that were i.p. infected with 5×10^3^ sfu of an *O*. *tsutsugamushi* Karp inoculum 6 hours later.

### ELISA

Concentrations of IFN-γ in serum were measured by ELISA, using the R&D DuoSet (R&D Systems, Minneapolis, USA), following the manufacturer’s protocol.

### Blood biochemistry

Serum ALT (alanine aminotransferase) and AST (aspartate aminotransferase) activities were measured using commercially available colorimetric assays (Reflotron, Roche Diagnostics, Mannheim, Germany).

### Histology and immunohistochemistry

Liver tissue was fixed in 4% formalin. Lungs were perfused with PBS through the pulmonary artery, filled with 4% formalin through the trachea and fixed in 4% formalin overnight. Embedding, sectioning and staining was performed at the mouse pathology core facility, University Hospital Hamburg Eppendorf, following standard procedures. For histology and immunohistochemistry (IHC) stains, standard methods were used. For immunofluorescence imaging, specimens were frozen in cryopreservation medium (TissueTek O.C.T Compound, Sakura Finetek, Torrance, USA) after fixation. Samples were sequentially reacted with rabbit anti-IBA1 (ionized calcium binding adapter molecule 1; Wako, Neuss, Germany) overnight; AlexaFluor488 donkey-anti-goat; 2F2 mAb; AlexaFluor594 donkey-anti-mouse (Life Technologies, Darmstadt, Germany). DAPI (Sigma, Germany) was used for nucleus counterstains (see [1] for details). Sections were embedded in Fluoromount G (Southern Biotech, Birmingham, USA) and viewed with a BZ-9000 Keyence fluorescence microscope or an Olympus confocal microscope.

For improved visibility of tissue lesions, nonlinear adjustment of the red color channel was applied in H&E stained liver sections of CD1d^-/-^ mice and their wildtype controls using the software GIMP (version 2.8). For quantification of IHC-data, pictures of at least 4 representative regions per individual organ were taken. Areas positive for the specific stain and total tissue areas excluding air lumen were quantified with the software ImageJ (ImageJ 1.48v, National Institutes of Health, USA).

### Statistical analysis

Data were analyzed using the Graphpad Prism 5.0 software. Descriptive statistics show mean +/- SD. Hypotheses were tested by two-tailed t test, by one-way or two-way analysis of variance (ANOVA) with Bonferroni post correction, or by Mantel-Cox test. A p value of <0.05 was considered significant.

### Ethics statement

Animal experimentation was conducted in agreement with the German animal protection law (TierSchG) and the Regulation for the protection of experimental animals (TierSchVersV). The experimental protocols have been reviewed and approved by the responsible Authority for Health and Consumer Protection, Department for Food and Veterinary Health of the State of Hamburg, Germany (approval number 74/09).

## Results

### CD8+ T cells are mandatory for the control of acute *O*. *tsutsugamushi* infection in BALB/c mice

**Fig 1:**
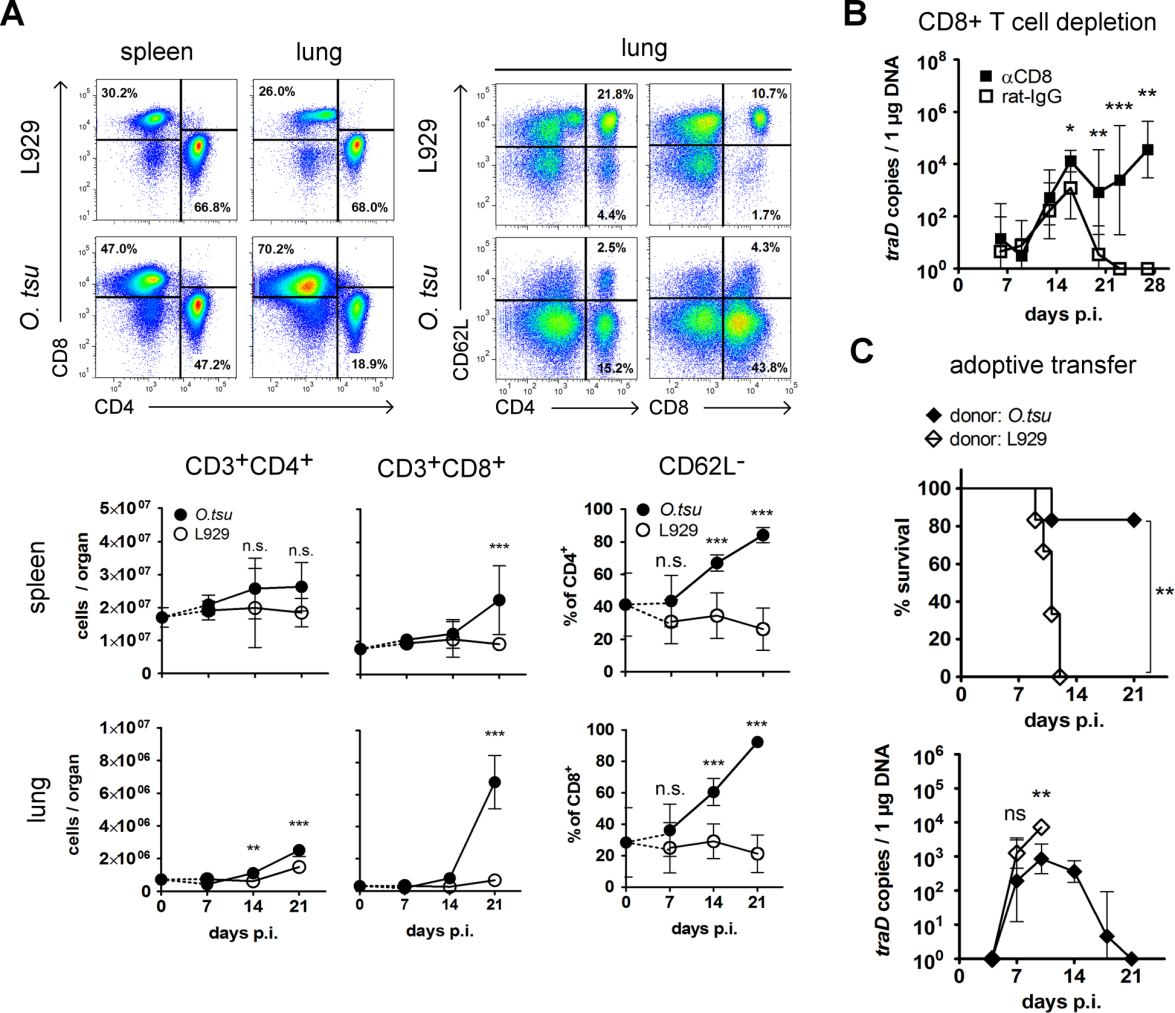
CD8+ T cells are activated in acute *O*. *tsutsugamushi* infection in BALB/c mice and protect against lethal i.p. infection. **A,** BALB/c mice were footpad-infected with *O*. *tsutsugamushi*. The numbers of CD3+CD4+ and CD3+CD8+ cells in spleen and lung as well as the percentage of CD62L- cells in the lung were measured by FACS. Dotplots show representative data of *O*. *tsutsugamushi*-infected mice (lower panels) or mock-controls (upper panels) at 21 days p.i. Graphs show mean values ± SD of pooled data from two independent experiments (n = 6). Values of untreated mice (d 0) are shown as reference for infected and mock-treated mice and were ignored for statistical analysis. Infected mice were compared to mock controls by two-way ANOVA with Bonferroni’s posttest. **B,** BALB/c mice were depleted of CD8+ T cells by injections of 0.5 mg anti-CD8 antibody or mock-treated with rat IgG at days -1; 6; 13; 20 p.i. Bacteremia was measured at indicated time points by *traD* qPCR. Shown are mean values ± SD of pooled data from two independent experiments (n = 8–9). CD8-depleted mice were compared to non-depleted mice by student’s t-tests. **C,** CD8+ T cells were purified from total splenocytes of infected or mock-treated BALB/c mice 21 days p.i. by magnetic cell separation. 1×10^7^ purified CD8+ T cells were adoptively transferred i.v. into BALB/c recipients that were challenged with *O*. *tsutsugamushi* i.p. 6 hours later. Graphs show survival (lower graph) and bacteremia (upper graph) of i.p. challenged recipients. Shown are mean values ± SD of pooled data from two independent experiments (n = 6). Survival curves were compared by Mantel-Cox test. The bacterial burden of recipients of CD8+ T cells from *O*. *tsutsugamushi*-infected or mock-treated donors was compared at individual time points by student’s t-tests. **A-C,** n.s. not significant; * p<0.05; ** p<0.01; *** p<0.001

We showed previously that BALB/c mice infected with *O*. *tsutsugamushi* Karp via the footpad developed increasing bacterial burdens in a large variety of organs during the first two weeks p.i., followed by a sharp decrease in the third week. The highest pathogen burden was found in the lung. The delayed pathogen control and the observation of high numbers of CD3+ cells in lung histology suggested a role of T cells in the protective immune response against *O*. *tsutsugamushi* in our model [1]. We therefore analyzed the size of CD4+ and CD8+ T cell populations and downregulation of CD62L as marker of activation not only in the spleen but also in the lung as a specific target tissue, which harbors only few lymphocytes under steady state conditions. Fig 1A shows that both T cell populations increased only marginally during the first two weeks, but during the third week p.i. CD8+ T cells expanded by 2-fold in the spleen and by 9-fold in the lung. A significant downregulation of CD62L on CD4+ and CD8+ T cells was noted already at 14 days p.i., and the percentage of CD62L- cells further increased to almost 100% at 21 days p.i., demonstrating a strong activation of T cells.

To investigate whether CD8+ T cells have a crucial role in infection control, we depleted BALB/c mice of CD8+ T cells by injection of a monoclonal antibody, or used IgG as a control. While bacteremia was similar in CD8+ T cell-depleted and control animals until day 14 p.i., it increased significantly and with a continuous tendency in depleted mice from 16 days p.i. on (Fig 1B). Thus, CD8+ T cells are required for limitation of bacterial growth during the third week p.i.

To demonstrate that primed CD8+ T cells are sufficient to mediate protection against *O*. *tsutsugamushi*, we adoptively transferred 10^7^ CD8+ T cells that were purified from the spleens of convalescent, footpad-infected BALB/c mice, 21 days p.i., into naïve BALB/c recipients. Recipients were challenged by i.p. infection with *O*. *tsutsugamushi* 6 hours later (Fig 1C). 84% of the recipients of CD8+ T cells from *O*. *tsutsugamushi*-infected mice were protected while all recipients of control CD8+ T cells died (Fig 1C, upper graph). An infection with measurable bacteremia at day 7 p.i. developed in both groups, but a significantly lower bacteremia was seen in recipients of experienced CD8+ T cells at 10 days p.i. The bacteremia declined to undetectable levels at day 21 p.i. (Fig 1C, lower graph).

We conclude that CD8+ T cells are indispensible for the control of *O*. *tsutsugamushi* growth in the acute phase of the infection, and that CD8+ T cells from *O*. *tsutsugamushi* infected mice are sufficient to protect against a lethal outcome in an otherwise naïve, immunocompetent recipient. However, the transfer of CD8+ T cells did not fully prevent the systemic spread of *O*. *tsutsugamushi* during the acute stage of infection.

### CD8+ T cells contribute to the control of *O*. *tsutsugamushi* during post-acute latency in C57BL/6 mice

**Fig 2:**
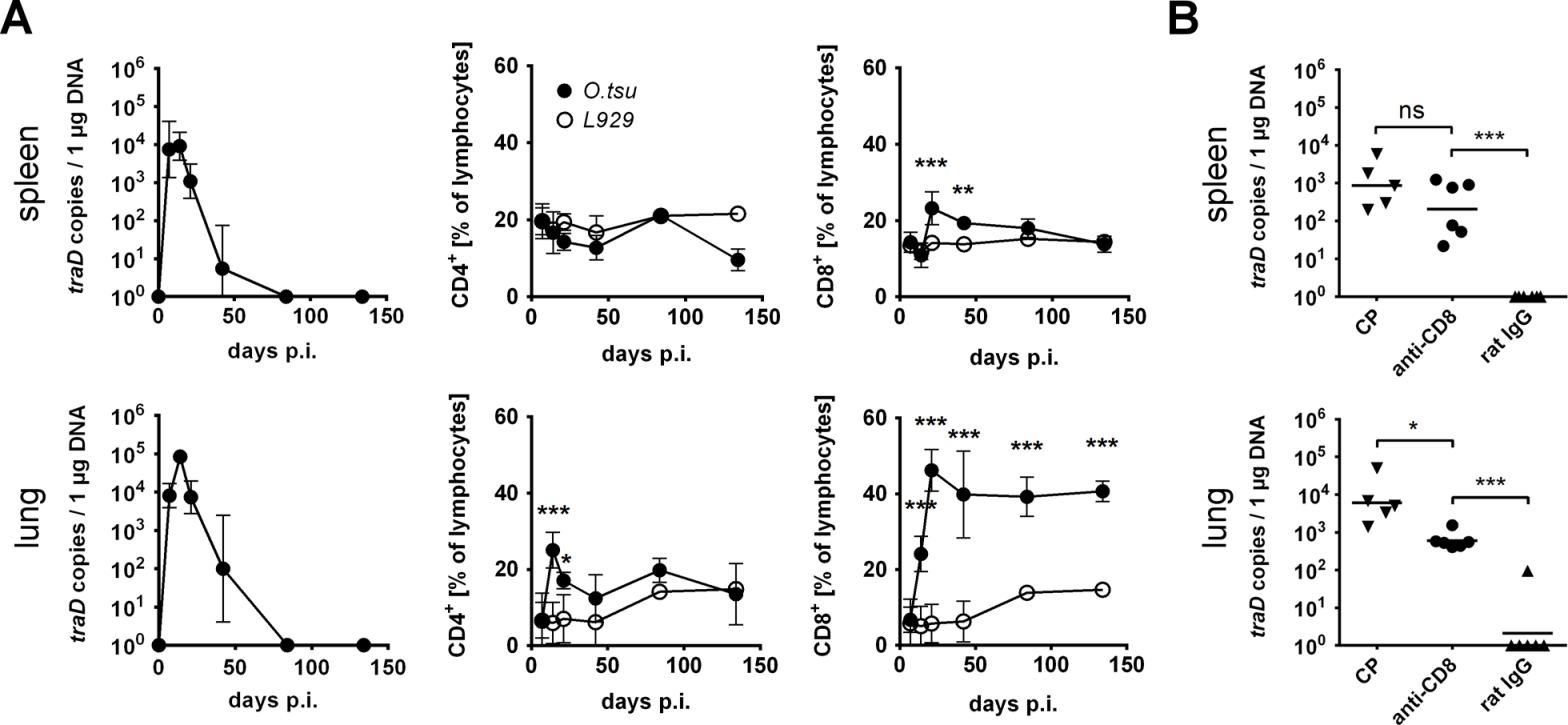
CD8+ T cells contribute to the control of *O*. *tsutsugamushi* during postacute latency in C57BL/6 mice. C57BL/6 mice were footpad-infected with *O*. *tsutsugamushi* or mock-infected. **A,** At the indicated time points, mice were sacrificed, and the bacterial burdens in spleen and lung were determined by qPCR (left panels). Additionally, the percentage of CD4+ and CD8+ T lymphocytes in spleen and lung were analyzed by FACS (middle and right panels). Shown are pooled data from two independent experiments (1^st^ exp., days 7 to 134: *O*. *tsu*-inf: n = 4 [n = 2 for FACS data on day 134], L929 controls: n = 2; 2^nd^ exp. days 7 to 42: n = 3). *O*. *tsutsugamushi*-infected mice were compared to mock-controls by two-way ANOVA with Bonferroni’s posttest. **B,** 84 days p.i., mice were treated with 8 mg CP, 0.5 mg anti-CD8 antibody or 0.5 mg rat IgG. A second treatment with 4 mg CP was performed 4–5 days later. 7 days later (91 days p.i.), the mice were sacrificed and the bacterial burdens in spleen (upper graph) and lung (lower graph) were measured by *traD* qPCR. Shown are pooled data from two independent experiments (n = 5–6). Anti-CD8 treated groups were compared to rat-IgG controls by one-way ANOVA with Bonferroni‘s posttest. CP: Cyclophosphamide. **A, B,** * p<0.05; ** p<0.01; *** p<0.001

Next, we investigated when *O*. *tsutsugamushi* was eliminated entirely from lung and spleen after acute infection, and whether changes in CD4+ and CD8+ lymphocyte compartments would be resolved upon clearance in these organs. After the peak at day 14 p.i., bacterial DNA burdens in spleen and lung as measured by qPCR decreased to undetectable levels at day 84 p.i. and were still below detection limit on day 135 p.i. (Fig 2A, left graphs).

In the spleen, the percentage of CD4+ T cells was constant, while an increased percentage of CD8+ T cells was found in the convalescence phase on days 21 and 42 p.i. In the lung, changes in the CD4+ T cell compartment were found only during the acute phase on days 14 and 21 p.i., but percentages of CD8+ T cells rose from day 14 p.i. and reached 45% at day 21 p.i. compared to 5% in the mock-controls. Interestingly, CD8+ T cells in the lung remained at percentages >40% during the whole observation period until day 135 p.i. (Fig 2A, middle and right graphs).

Previous reports showed that unspecific immunosuppression with CP could reactivate latent infection with *O*. *tsutsugamushi* in mice [20]. Our results suggested that long-term expansion of CD8+ T-cells could be required for the containment of *O*. *tsutsugamushi* during the latent phase. We thus depleted CD8+ T cells with a monoclonal antibody in infected mice at 84 days p.i., when they had overcome the symptomatic acute phase (Fig 2B). CP treatment was used as a control. Rat IgG was administered in non-depleted controls. No bacterial DNA was measured 7 days post treatment in rat IgG-treated mice in the spleen and in five of six mice in the lung (1 mouse exhibited low level infection). In contrast, more than 100 *traD* copies/1 μg DNA were found in CD8+ T cell-depleted mice in spleen and lung. These data suggest that an expanded population of CD8+ T cells is necessary to prevent recurrent growth of *O*. *tsutsugamushi* during the latent phase of infection. However, compared to CD8-depleted mice, CP-treated mice had 4 times higher bacterial burden in the spleen and 12 times higher bacterial burden in the lung. Since less CD8+ T cells were found in the peripheral blood after antibody-dependent depletion than after CP treatment (S2 Fig), this observation could not be attributed to insufficient depletion efficiency. Instead, we hypothesize the existence of CD8+ T cell-independent immune mechanisms that further contribute to *O*. *tsutsugamushi* control in the post-acute phase.

### Genetically CD8+ T cell deficient β_2_m^-/-^ mice develop a strong IFN-γ response but fail to control *O*. *tsutsugamushi* infection

**Fig 3:**
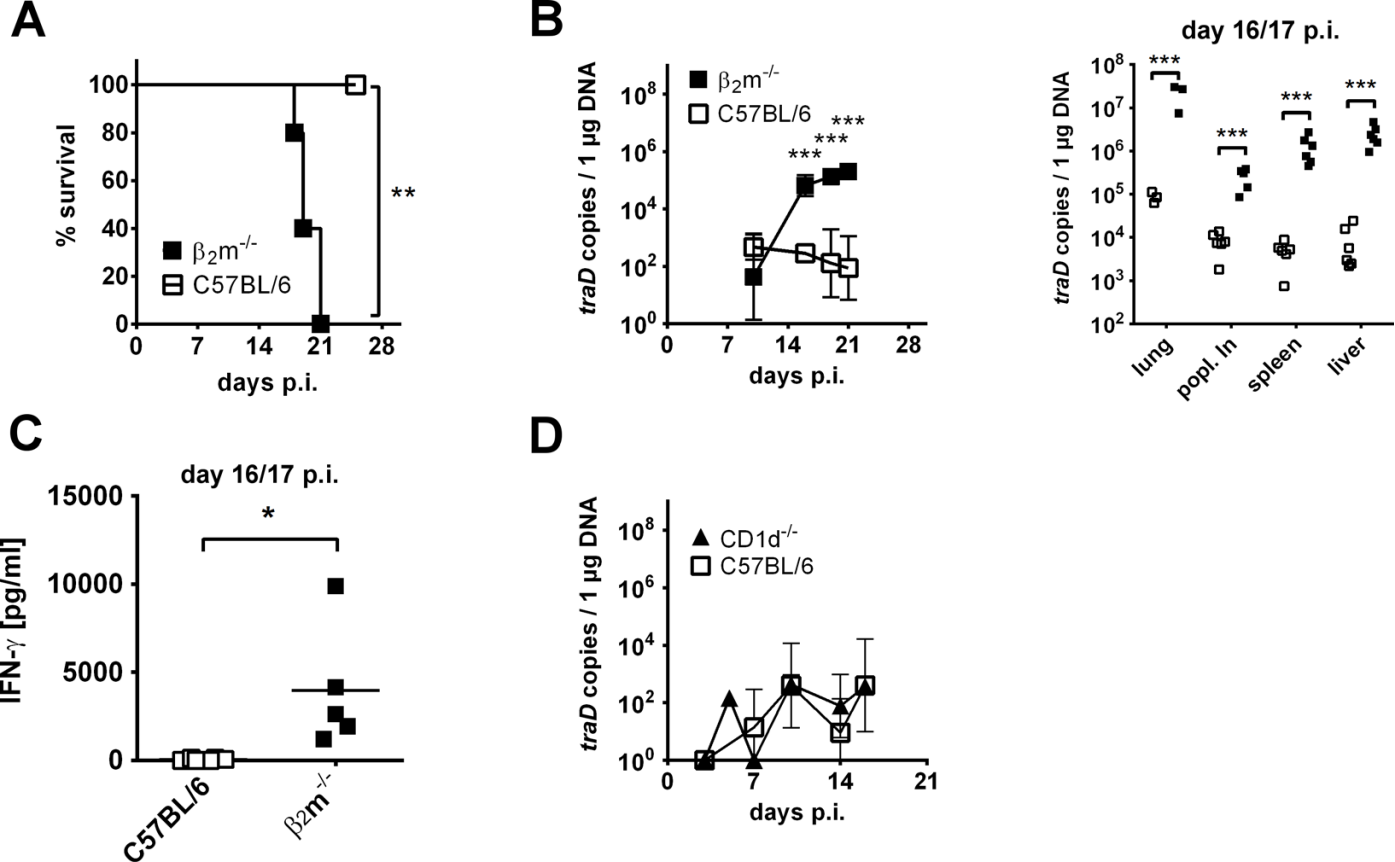
C57BL/6 wildtype and CD1d^-/-^ but not CD8+ T cell deficient β_2_m^-/-^ mice control *O*. *tsutsugamushi* infection. **A-C,** β_2_m^-/-^ mice or C57BL/6 controls were footpad-infected with *O*. *tsutsugamushi* without further treatment. **A,** Survival curve. Data from one of two representative experiments (n = 5). Significance was determined by Mantel-Cox test. **B,** Bacteremia at indicated time points (left) and *O*. *tsutsugamushi* burdens in target organs at days 16 or 17 p.i. (right) are depicted. Shown are mean values ± SD from one of two representative experiments (bacteremia, n = 5) or pooled data from two independent experiments (organ loads, mean ± SD, n = 6, lung: n = 3). β_2_m^-/-^ mice were compared to C57BL/6 controls by student’s t-tests. **C,** IFN-γ was measured by ELISA in serum from β_2_m^-/-^ mice or C57BL/6 controls at days 16 or 17 p.i. IFN-γ levels were compared by student’s t-test. **D,** CD1d^-/-^ mice or C57BL/6 controls were footpad-infected with *O*. *tsutsugamushi*. Bacteremia was measured at indicated time points by qPCR. **A-D,** * p<0.05; ** p<0.01; *** p<0.001.

To further demonstrate the importance of CD8+ T cells in *O*. *tsutsugamushi* infection, we investigated the infection course in a mouse model that is genetically deficient of CD8+ T cells on the C57BL/6 background. β2-microglobulin knockout (β_2_m^-/-^) mice do not express functional MHC-I molecules and are CD8+ T cell deficient due to a lack of positive selection in the thymus [45]. We infected β_2_m^-/-^ mice by footpad inoculation with the Karp strain of *O*. *tsutsugamushi*. 100% of these mice had succumbed to the infection by 21 days p.i., while all C57BL/6 wildtype controls survived (Fig 3A). This suggests that no other cell type could compensate a lack of CD8+ T cells. In β_2_m^-/-^ mice we found more than 100-fold higher bacteremia between days 16 and 21 p.i., compared to the wildtype (Fig 3B, left), and lung, liver, popliteal lymph node and spleen of β_2_m^-/-^ mice had significantly higher bacterial burdens on day 16 or 17 p.i. (Fig 3B, right).

IFN-γ is regarded as a hallmark of anti-*O*. *tsutsugamushi* immunity [1, 8, 9, 46]. However, while serum IFN-γ was low in *O*. *tsutsugamushi* infected C57BL/6 wildtype controls (mean 50 pg/ml), 80-fold higher concentration (mean 4 ng/ml) was measured in infected β_2_m^-/-^ mice 16 or 17 days p.i. (Fig 3C). This suggests that although production of IFN-γ is massively increased in the absence of functional CD8+ T cells, IFN-γ-dependent effector mechanisms are insufficient to provide protection.

The CD1d molecule, which is the restriction element of NKT cells, consists of a β_2_m subunit. Thus, β_2_m^-/-^ mice lack, besides CD8+ T cells, also NKT cells [47, 48], a cell type with potent anti-bacterial mechanisms that is most abundant in the liver. To control the influence of NKT cells on *O*. *tsutsugamushi* infection, we infected CD1d^-/-^ mice. We did not observe differences in bacteremia between infected CD1d^-/-^ mice and their C57BL/6 wildtype controls (Fig 3D). It is known that, besides CD8+ T cell and NKT cell deficiency, β2m^-/-^ mice also have defects in iron homeostasis [49] that might influence replication of intracellular bacteria. However, we used CD8+ T cell depletion as an independent method to demonstrate CD8+ T cell-mediated protection (Fig 1B). We thus conclude that CD8+ and not NKT cells are required for efficient growth control of *O*. *tsutsugamushi*.

Accordingly, we showed in two genetically different mouse strains by two different approaches that CD8+ T cells are indispensable for the immunological control during the late acute and the latent phase in a mouse footpad infection model using the Karp strain of *O*. *tsutsugamushi*.

### CD8+ T cells cause hepatocellular injury followed by macrophage organization

**Fig 4:**
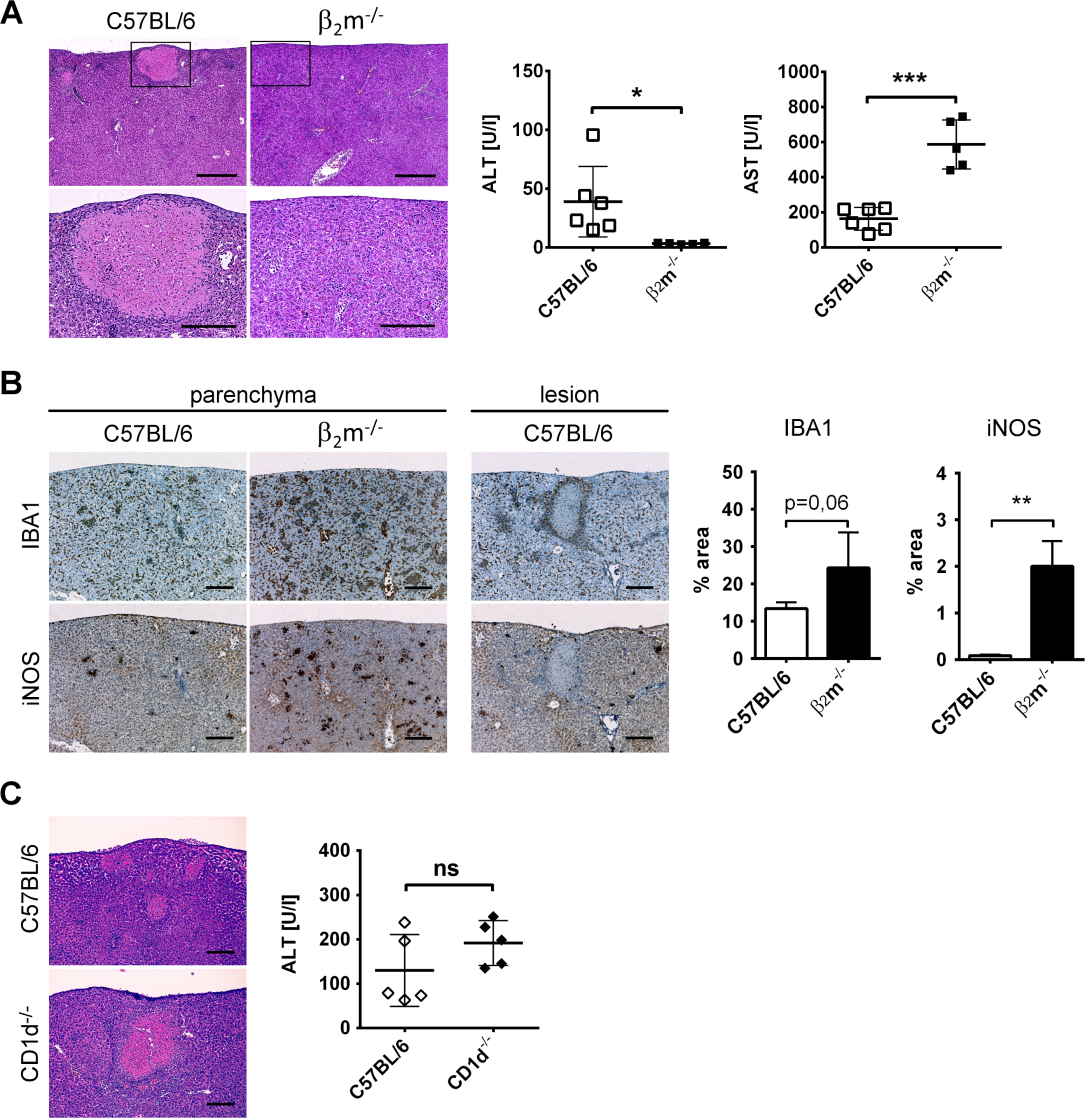
Hepatocellular injury is mediated by CD8+ T cells and triggers macrophage organization. **A-B,** β_2_m^-/-^ mice or C57BL/6 controls were footpad-infected with *O*. *tsutsugamushi*. 16 days p.i. the mice were sacrificed. **A,** H&E stained liver sections. Rectangles in upper panels indicate zones of higher magnification (lower panels). Scale bars, upper panels: 500 μm, lower panels: 200μm. Images show representative regions. The graphs show serum ALT (left) or serum AST (right) levels. Data are pooled from two independent experiments (n = 6) and analyzed by student’s t-tests. **B,** Consecutive liver sections stained with antibody against IBA1 (upper panels) or iNOS (lower panels). Scale bars: 200 μm. Images show representative regions. Graphs show percentages of stain^+^ areas per total tissue area in the parenchyma. For quantification 4 representative regions were chosen from each mouse (n = 3). **C,** CD1d^-/-^ mice or C57BL/6 controls were footpad-infected with *O*. *tsutsugamushi*. 16 days p.i. the mice were sacrificed. Images show representative regions of H&E stained liver sections (n = 5). Scale bars: 200 μm. The graph shows serum ALT levels (means +SD, n = 5). Data were analyzed by student’s t-tests. **A-C,** ns: not significant; * p<0.05; ** p<0.01; *** p<0.001.

We next wanted to know whether, besides increasing resistance to infection, CD8+ T cells can cause or protect from infection-induced organ pathology. To investigate the role of CD8+ T cells in hepatic inflammation and tissue damage, we analyzed liver samples from wildtype and β_2_m^-/-^ mice 16 days p.i. by histopathology. In wildtype mice, subcapsular necrotic lesions were found that were completely absent in β_2_m^-/-^ mice (Fig 4A, left panels). Serum activity of ALT, which specifically indicates hepatocyte injury, was also elevated only in wildtype but not β_2_m^-/-^ mice (Fig 4A, middle panel). Activity of AST, which is not hepatocyte-specific and may be derived from other tissues [50], was increased in β_2_m^-/-^ mice (Fig 4A, right panel). Thus, CD8+ T cells are mediators of hepatocyte injury during acute *O*. *tsutsugamushi* infection, as demonstrated by histopathology and elevation of serum ALT.

We observed that IBA1+ macrophages clustered in nodules in both wildtype and β_2_m^-/-^ mice (Fig 4B, left and middle panels), similar to what was found in BALB/c mice [1]. While there was a tendency to stronger infiltration with IBA1+ macrophages (p = 0.06) in β_2_m^-/-^ mice compared to the wildtypes, the expression of iNOS was significantly higher (Fig 4B). CD8+ T cell deficiency therefore increased the degree of macrophage infiltration and activation. We also observed in the liver of C57BL/6 wildtype mice that IBA1+iNOS+ and IBA1+iNOS- macrophages organized around the necrotic lesions (Fig 4B, right panels). Thus, CD8+ T cells caused limited hepatocyte injury in the liver and triggered the formation of necrotic, macrophage-walled structures. Despite stronger invasion of macrophages in β_2_m^-/-^ mice, possibly a reaction to the increased bacterial burden, the infection could not be controlled in the absence of CD8+ T cells.

CD1d-restricted NKT cells, which are also lacking in β_2_m^-/-^ mice, constitute 20–30% of murine hepatic lymphocytes [51] and, in some infections, play an important role in the early antimicrobial immune response [52]. In order to exclude a role for NKT cells in liver pathology, we analyzed liver and serum samples of *O*. *tsutsugamushi*-infected CD1d^-/-^ mice on day 16 p.i. Necrotic lesions were also observed in livers of CD1d^-/-^ mice (Fig 4C, left panels). Moreover, serum ALT activity was also elevated in CD1d^-/-^ mice and did not differ significantly from the wildtypes, which demonstrates that NKT-cells are not responsible for the development of hepatocyte injury and necrotic liver lesions during murine *O*. *tsutsugamushi* infection.

### CD8+ T cells limit pathogen burden in the lung and cause macrophage infiltration into pulmonary BALT

**Fig 5:**
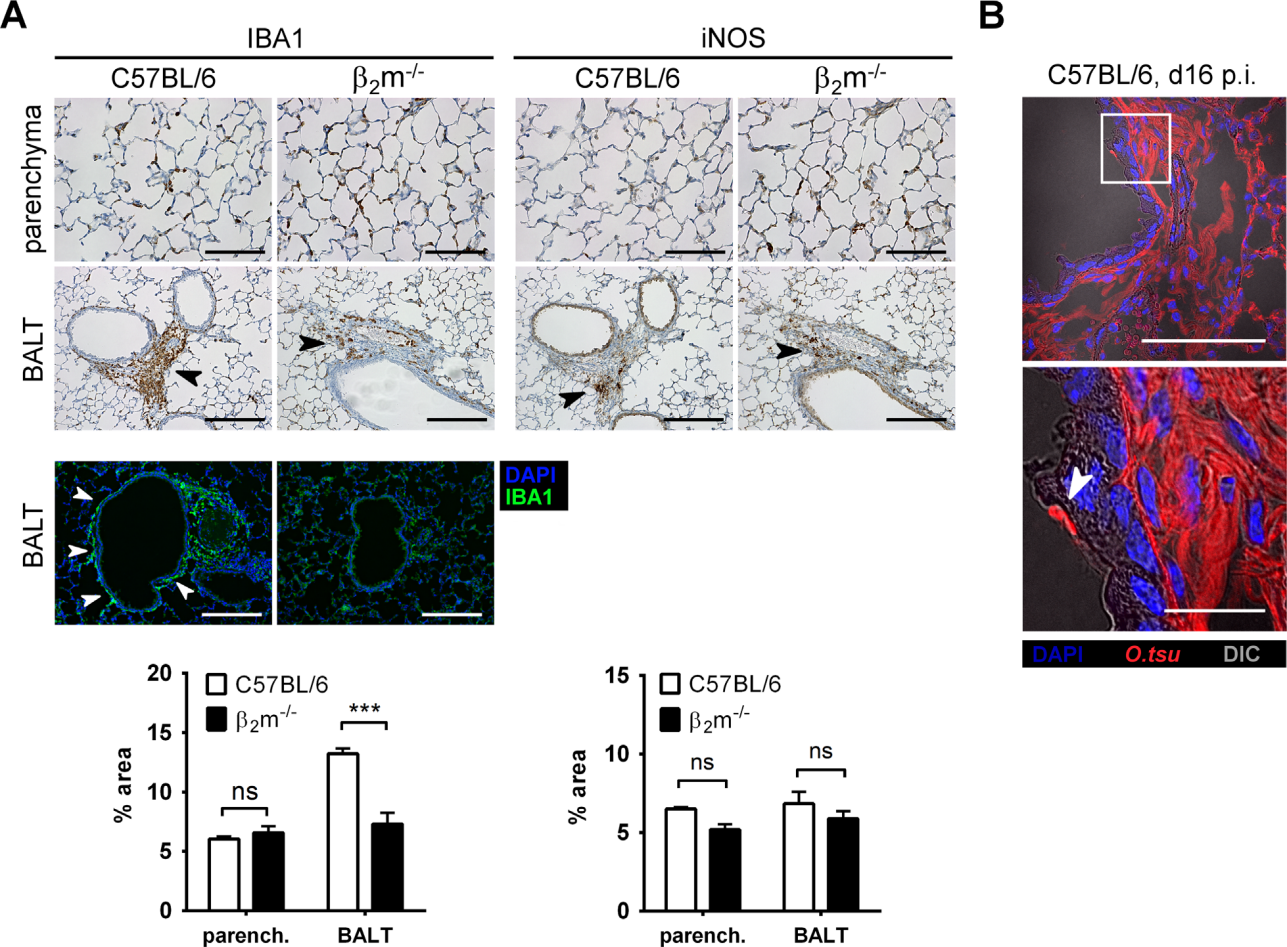
Decreased macrophage recruitment to pulmonary BALT in β_2_m^-/-^ mice. β_2_m^-/-^ mice or C57BL/6 controls were footpad-infected with *O*. *tsutsugamushi*. 16 or 17 days p.i., mice were sacrificed. **A,** Consecutive lung sections, stained for IBA1 or iNOS (top, middle) and cryosections, stained for IBA1 (green) and DAPI (blue) (bottom). Arrowheads indicate IBA1+ or iNOS+ cells in BALT regions (immunohistochemistry) or IBA1+ cells at peribronchial lining (immune fluorescence). Scale bars, parenchyma: 100 μm; BALT: 200 μm. Images show representative regions. Graphs show percentages of stain^+^ areas per total tissue area from 4 representative regions of each mouse (n = 3). β_2_m^-/-^ were compared to C57BL/6 controls by two-way ANOVA with Bonferroni’s posttest. ns: not significant; *** p<0.001 **B,** Immunofluorescence of bronchiolar lining, stained with antibody against the *O*. *tsutsugamushi* 56 kD antigen (red) and DAPI (blue) overlayed with DIC. Images were captured with an Olympus confocal microscope. Bottom panel shows region of higher magnification. Arrowhead indicates a bacterium at the luminal side inside an epithelial cell. Scale bars: 100 μm (overview); 20 μm (detail). parench.: parenchyma.

We previously presented evidence that infected IBA1+ macrophages accumulated in the lung parenchyma and pulmonary bronchus-associated lymphatic tissue (BALT) [1]. To investigate whether CD8+ T cells shape the pulmonary macrophage response, we investigated the distribution of IBA1+ macrophages and the induction of iNOS in lungs of β_2_m^-/-^ and C57Bl/6 mice by histopathology. While in the lung parenchyma, the percentage of IBA1+ macrophages did not differ between β_2_m^-/-^ and C57BL/6 wildtype mice, less IBA1+ macrophages were found in BALT areas of β_2_m^-/-^ compared to C57BL/6 wildtype mice (Fig 5A, left panels). The production of iNOS in the lungs of β_2_m^-/-^ mice did not differ significantly at both locations (Fig 5A, right panels). This indicates a contribution of CD8+ T cells to macrophage recruitment to BALT areas in C57BL/6 wildtype mice, while the parenchymal infiltration of macrophages is independent of CD8+ T cells.

As shown more clearly by immunofluorescence of C57BL/6 wildtype lungs, macrophages do not only cluster perivascularly, but form a slender cellular cuff around the bronchial epithelium in the wildtypes (Fig 5A, lower panels). We show here that bronchial epithelial cells were infected with *O*. *tsutsugamushi* (Fig 5B). Macrophage-rich BALTs and peribronchial macrophage linings may thus form in response to a CD8+ T cell reaction against infected epithelial cells. However, further studies have to be performed to uncover the mechanisms of *O*. *tsutsugamushi* control in this compartment.

## Discussion

A protective role of T cells in anti-*O*. *tsutsugamushi* immunity has been suggested by several studies [8, 53, 54], but direct experimental evidence for protection by the CD8+ subset of T cells has been lacking so far. CD8+ T cells may generally be more important for the immunological defense than CD4+ T cells in many rickettsial infections [46, 55–57], but since *O*. *tsutsugamushi* is unique among the Rickettsiaceae regarding its morphology, physiology, and genome structure [58, 59], an independent investigation on the role of CD8+ T cells was warranted. Several studies reported expansion and activation of CD8+ T cells during *O*. *tsutsugamushi* infection in humans [15, 16], and our footpad inoculation mouse model recapitulates the increase of CD8+ T cells in the convalescent phase that is seen in these patients.

We demonstrated CD8+ T cell-dependent protection by showing uncontrolled bacterial growth in CD8+ T cell-depleted BALB/c mice as well as in CD8+ T cell-deficient β_2_m^-/-^ mice. Furthermore, the study provides evidence that CD8+ T cells are necessary to control *O*. *tsutsugamushi* in the persistent phase. We could also show that adoptively transferred CD8+ T cells from *O*. *tsutsugamushi*-infected mice protected i.p.-challenged BALB/c recipients from an otherwise lethal outcome. As a potential limitation to this conclusion it has to be noted that the transferred population showed a content of <5% CD4+ T cells. We cannot exclude that the presence of lymphocyte subsets other than CD8+ T cells in this population was functionally relevant. However, the high number of CD8+ T cells makes it highly likely that the protective effect was attributable to the CD8+ T cell subset. Clearly, studies on the contribution of CD4+ T cells or NK cells to protection against *O*. *tsutsugamushi*, by depletion and/or adoptive transfer, are needed, but this question was beyond the scope of the present study. In summary, we concluded that CD8+ T cells are essential for protective immunity against *O*. *tsutsugamushi*.

However, even experienced CD8+ T cells were not sufficient to fully prevent bacterial proliferation in wildtype mice. Moreover, CD8+ T cells were not sufficient to completely eradicate the pathogen from the body, as demonstrated by CD8+ T cell depletion during persistence. This finding is of vital impact for the design of an *O*. *tsutsugamushi* vaccine: A vaccination strategy based on CD8+ T cells alone may not be able to prevent low-level infection and persistence of *O*. *tsutsugamushi* [18, 20]. To achieve the goal of sterilizing immunity, CD4+ T cell or antibody epitopes may have to be included [60].

We could demonstrate that CD8+ T cells were protective in persistent *O*. *tsutsugamushi* infection, however immunosuppression with CP had an even larger effect on the bacterial burden in the lung during persistence compared to depletion of CD8+ T cells. This was not due to an insufficient antibody-dependent depletion of CD8+ T cells (S2 Fig). Since CP is active in a wide variety of immune cells including B cells, T cells and hematopoietic progenitor cells [22–25], we hypothesize the existence of additional CD8+ T cell-independent cellular mechanisms that contribute to the containment of persistent *O*. *tsutsugamushi*. CD4+ T cell- [61] or NK cell-derived cytokines could exert this additional effect.

IFN-γ is regarded as a hallmark of anti-*O*. *tsutsugamushi* immunity, and increased IFN-γ serum levels have been found in scrub typhus patients [35, 62, 63] as well as in mice after antigen provocation or i.p. challenge [10, 11]. Our data show surprisingly low concentrations of serum IFN-γ in wildtype control mice (mean of 52.6 pg/ml) at day 16/17 p.i. Early studies had shown that serum IFN-γ concentrations can change dramatically within just a few days [11]. Consistent with these findings, IFN-γ serum levels during the course of a primary infection in mouse models correlated with *O*. *tsutsugamushi* burden in blood as well as in target organs [1, 9]. Since our measurements were performed on day 16/17 p.i., which is after the peak of bacteremia (Fig 3B), IFN-γ concentrations in wildtype mice may have already declined from their maxima. In infected β_2_m^-/-^ mice however, we observed elevated serum IFN-γ levels (Fig 3C) that reflect the high pathogen burden at that time (Fig 3B). Therefore we suggest that IFN-γ serum levels correlate with *O*. *tsutsugamushi* bacteremia, and that IFN-γ dependent effector mechanisms alone are insufficient to mediate protection against *O*. *tsutsugamushi* Karp infection *in vivo*.

A common observation in scrub typhus patients is elevation of liver transaminases [64], and hepatic lesions have been described in *O*. *tsutsugamushi*-infected mice and humans [4, 65]. Liver failure is even a frequent cause of death in fatal scrub typhus cases. As shown here, hepatic necroses and elevation of serum ALT was only observed in *O*. *tsutsugamushi*-infected C57BL/6 wildtype but not in CD8+ T cell-deficient β_2_m^-/-^ mice. We thus provide evidence that liver pathology is caused by the CD8+ T cell-dependent immune response and not by the pathogen itself. We successfully controlled for the influence of NKT cells by demonstrating that the phenotype of CD1d^-/-^ mice was comparable to wildtype mice. Thus, both protective role and pathogenic function could be attributed to CD8+ T cells rather than NKT cells. This is in contrast to a previous report that claimed a direct cytopathic effect of *O*. *tsutsugamushi* in infected hepatocytes [65]. We suggest that cellular cytotoxicity by CD8+ T cells is the most important effector mechanism for pathogen control and development of liver injury during *O*. *tsutsugamushi* infection. Experimental limitations prohibited a comprehensive study of both aspects in our model: Perforin^-/-^ mice showed no difference in bacteremia, but increased organ loads at day 11 p.i. However, according to ethical guidelines, mice had to be sacrificed between days 10 and 13 p.i., i.e. before the onset of measurable liver injury in control mice (S4 Fig).

It remains of interest whether CD8+ T cells are sufficient to prevent pathogen growth and to cause tissue injury in *O*. *tsutsugamushi* infection, or whether accessory cells are required to exert these effects. Mechanisms of CD8+ T cell-mediated liver immunopathology have been elucidated in more detail in viral hepatitis, such as hepatitis B virus (HBV) infection. It was shown in HBV models that although CD8+ T cells are able to directly lyse hepatocytes expressing cognate antigen [66], this recognition by CD8+ T cells is only the first step in a cascade of events that involves amplification of immunopathology by macrophages and neutrophils [67]. Similarly, CD8+ T cells were required for hepatocellular injury and activation of hepatic macrophages during Lassa virus infection [68]. CD8+ T cell dependent control of *Listeria monocytogenes* was shown to rely on CCL3 secretion by CD8+ T cells that drives antibacterial effector mechanisms in inflammatory monocytes and neutrophils [69, 70]. Our data presented here demonstrate that in the liver, CD8+ T cell-dependent hepatic necroses were surrounded by IBA1+ macrophages, while in the lung, accumulation of IBA1+ macrophages surrounding *O*. *tsutsugamushi*-infected bronchial epithelium equally depended on CD8+ T cells. Thus, a cooperation between CD8+ T cells and macrophages may also be of functional importance for protection or immunopathology in *O*. *tsutsugamushi* infection. Further studies have to provide more evidence for this hypothesis and decipher the provenance and role of hepatic perilesional and pulmonary BALT macrophages.

In the present study we could show that in the lung, an expanded CD8+ T cell population persisted after the acute infection had subsided. Moreover, CD8+ T cells were mandatory for pathogen control during persistent *O*. *tsutsugamushi* infection. The persistence of CD8+ T cells in target organs can also be seen in some virus infections. For example in hepatitis C virus (HCV) infection, an inverse relationship between intrahepatic CTL responses and viral load was shown, underlining the hypothesis that HCV-specific CTL limit viral replication in patients with chronic HCV infection [71]. In influenza virus-infected mice, antigen-specific CD8+ T cells were also found at high frequencies in the lungs several month after recovery [72].

In the present study it became evident that CD8+ T cells also cause tissue damage during *O*. *tsutsugamushi* infection. It is possible that the CD8+ T cell response needs to be attenuated by regulatory mechanisms in order to avoid excessive collateral tissue damage, with the trade-off that viable bacteria persist in the host. On the other hand, sustained surveillance of bacteria by a small number of persisting effector cells in infected tissues could be a tolerable side-effect. Also, the acceptance of a low-level persistence of pathogens might be a self-protecting mechanism allowing constant antigen-specific stimulation of the immune system to avoid waning immunity, like already proposed for LCMV infection [73].

Taken together, we report a number of novel aspects of *O*. *tsutsugamushi* infection in our murine model that closely approximates natural infection: we report for the first time (1) the kinetics of CD8+ T cell activation in lung and spleen by flow cytometry during acute infection, (2) the impact of CD8+ T cell deficiency on bacterial growth control and survival, (3) a prolonged expansion of CD8+ T cells during the latency phase that prevents reactivation of bacterial replication and (4) CD8+ T-cell-dependent liver and lung injury. Our study thus provides evidence that CD8+ T cells are non-redundant for the protection against *O*. *tsutsugamushi* during acute and persistent infection. However CD8+ T cells also contribute to liver pathology and influence the pattern and extent of macrophage recruitment in liver and lung tissue.

## Supporting Information

S1 Fig Efficiency of CD8+ T cell depletion during acute phase. Peripheral blood leukocytes of CD8+ T cell depleted or control mice were stained at indicated time points with anti-CD3 and anti-CD8. The percentage of CD8+ T cells was analyzed by flow cytometry.
(TIF)

S2 Fig Efficiency of CD8+ T cell depletion during persistent phase. Footpad-infected mice were treated with anti-CD8 monoclonal antibody, rat IgG, or CP at 84 days p.i. (see Fig 2 and methods for details). Peripheral blood leukocytes of CD8+ T cell-depleted, CP-treated, or control mice were stained at indicated time points with fluorescence-labeled anti-CD3, anti-CD4, anti-CD8, and anti-B220 antibodies and analyzed by flow cytometry. Representative plots show CD4+ and CD8+ populations in the CD3+B220- gate 7 days post treatment. Graphs show mean absolute cell numbers +/-SD of CD4+ and CD8+ T cells.
(TIF)

S3 Fig CD8+ T cells were purified by negative magnetic isolation. Total cells before (upper plot) and after (bottom plot) purification were stained with anti-CD3 and anti-CD8 antibodies and analyzed by flow cytometry. Plots show representative data and percentages show mean values of all samples that were used for adoptive transfer experiments (Fig 1).
(TIF)

S4 Fig Prf1-/- mice develop higher pathogen burden in target organs than C57BL/6 wildtype mice and succumb to *O*. *tsutsugamushi* infection before the onset of liver injury. Prf1^-/-^ mice or C57BL/6 controls were footpad-infected with *O*. *tsutsugamushi*. **A,** Survival curve. Shown are pooled data from two independent experiments (n = 10). Significance was determined by Mantel-Cox test. **B,** Bacteremia at indicated time points is depicted. Shown are pooled data from two independent experiments (n = 10, except deceased mice as shown in A). **C,** *O*. *tsutsugamushi* burdens in target organs at day 11 p.i. Shown are pooled data from two independent experiments (n = 6). Prf1^-/-^ mice were compared to C57BL/6 controls by two-way ANOVA. **D,** The graph shows serum ALT levels at day 11 p.i. from one experiment (means ± SD, n = 3–4). Data were analyzed by student’s t-test. **A-D,** ns: not significant; * p<0.05; ** p<0.01; *** p<0.001.
(TIF)
