# Supplementary figures and images for: Protective and Pathogenic Roles of CD8+ T Lymphocytes in Murine Orientia tsutsugamushi Infection
Source: PLoS Negl Trop Dis. 2016 Sep 8;10(9):e0004991. doi: 10.1371/journal.pntd.0004991 (PMC5015871; doi:10.1371/journal.pntd.0004991)

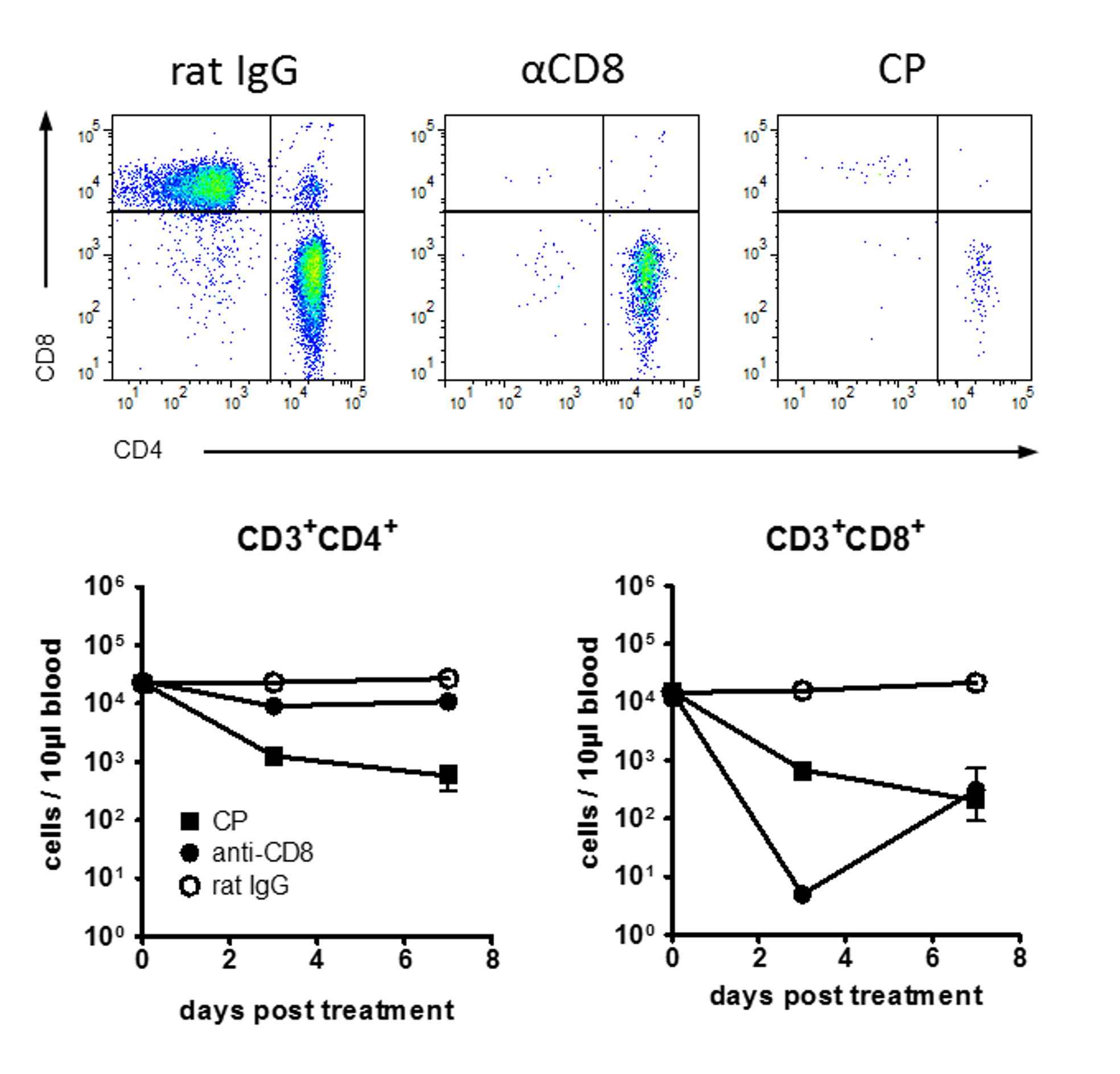

Supplement: S2 Fig — Footpad-infected mice were treated with anti-CD8 monoclonal antibody, rat IgG, or CP at 84 days p.i. (see Fig 2 and methods for details). Peripheral blood leukocytes of CD8+ T cell-depleted, CP-treated, or control mice were stained at indicated time points with fluorescence-labeled anti-CD3, anti-CD4, anti-CD8, and anti-B220 antibodies and analyzed by flow cytometry. Representative plots show CD4+ and CD8+ populations in the CD3+B220- gate 7 days post treatment. Graphs show mean absolute cell numbers +/-SD of CD4+ and CD8+ T cells. (TIF) [file pntd.0004991.s002.tif]

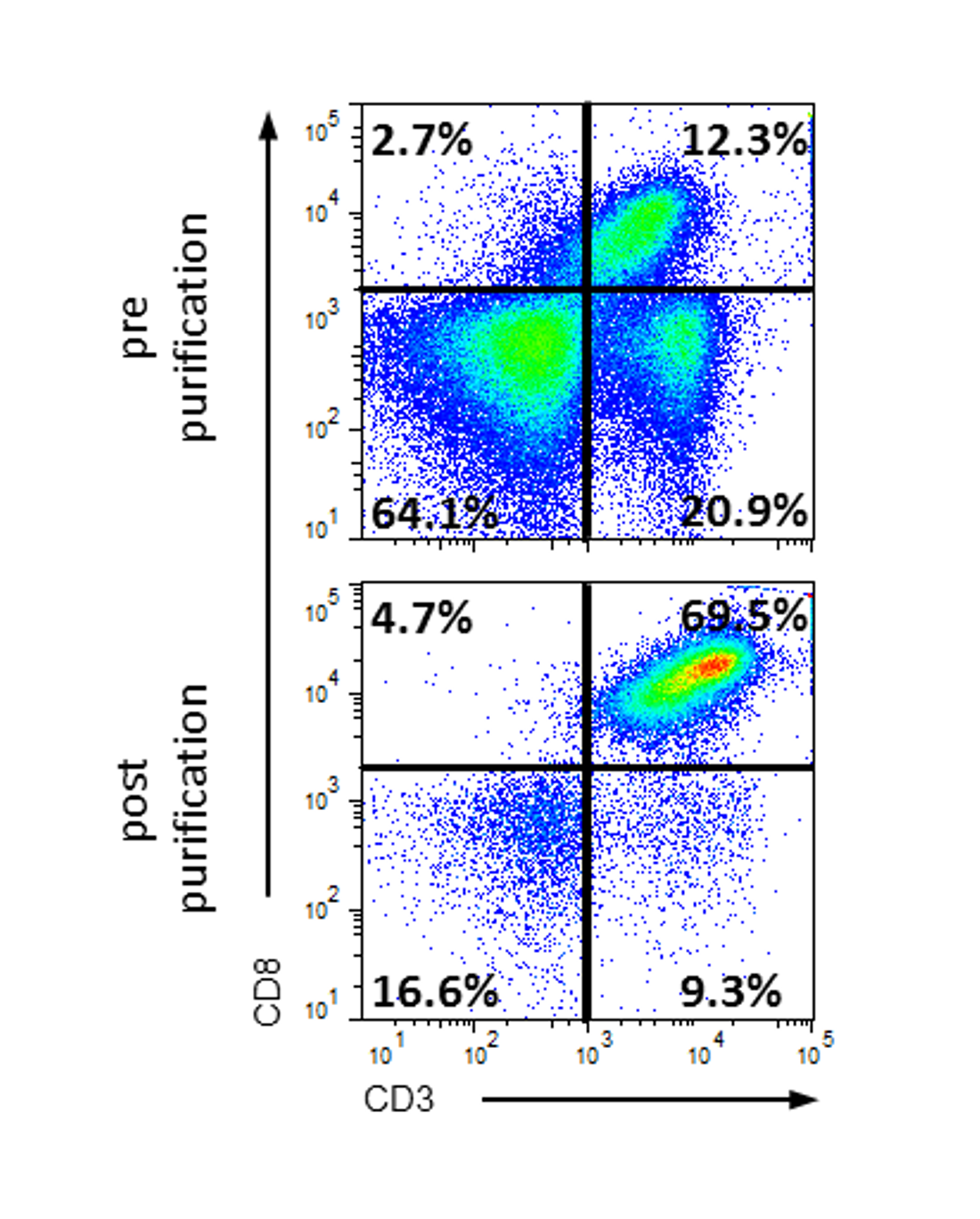

Supplement: S3 Fig — Total cells before (upper plot) and after (bottom plot) purification were stained with anti-CD3 and anti-CD8 antibodies and analyzed by flow cytometry. Plots show representative data and percentages show mean values of all samples that were used for adoptive transfer experiments (Fig 1). (TIF) [file pntd.0004991.s003.tif]

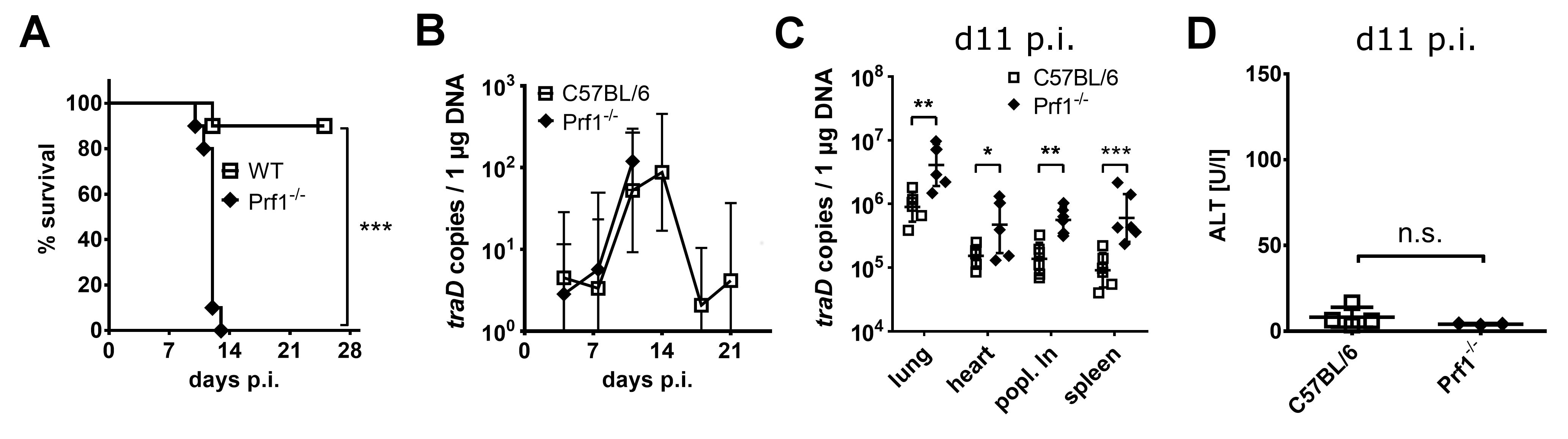

Supplement: S4 Fig — Prf1-/- mice or C57BL/6 controls were footpad-infected with O. tsutsugamushi. A, Survival curve. Shown are pooled data from two independent experiments (n = 10). Significance was determined by Mantel-Cox test. B, Bacteremia at indicated time points is depicted. Shown are pooled data from two independent experiments (n = 10, except deceased mice as shown in A). C, O. tsutsugamushi burdens in target organs at day 11 p.i. Shown are pooled data from two independent experiments (n = 6). Prf1-/- mice were compared to C57BL/6 controls by two-way ANOVA. D, The graph shows serum ALT levels at day 11 p.i. from one experiment (means ± SD, n = 3–4). Data were analyzed by student’s t-test. A-D, ns: not significant; * p<0.05; ** p<0.01; *** p<0.001. (TIF) [file pntd.0004991.s004.tif]
